# Alberta Stroke Program Early CT Score Infarct Location Predicts Outcome Following M2 Occlusion

**DOI:** 10.3389/fneur.2017.00098

**Published:** 2017-03-14

**Authors:** Muhib Khan, Grayson L. Baird, Richard P. Goddeau, Brian Silver, Nils Henninger

**Affiliations:** ^1^Neuroscience Institute, Division of Neurology, Spectrum Health, Grand Rapids, MI, USA; ^2^Michigan State University College of Human Medicine, Grand Rapids, MI, USA; ^3^Lifespan Biostatistics Core, Rhode Island Hospital, Providence, RI, USA; ^4^Department of Neurology, University of Massachusetts Medical School, Worcester, MA, USA; ^5^Department of Neurology, Warren Alpert Medical School of Brown University, Providence, RI, USA; ^6^Department of Psychiatry, University of Massachusetts Medical School, Worcester, MA, USA

**Keywords:** M2 occlusions, outcome, Alberta Stroke Program Early CT Score, stroke, thrombolysis

## Abstract

**Background:**

Although it is generally thought that patients with distal middle cerebral artery (M2) occlusion have a favorable outcome, it has previously been demonstrated that a substantial minority will have a poor outcome by 90 days. We sought to determine whether assessing the Alberta Stroke Program Early CT Score (ASPECTS) infarct location allows for identifying patients at risk for a poor 90-day outcome.

**Methods:**

We retrospectively analyzed patients with isolated acute M2 occlusion admitted to a single academic center between January 2010 and August 2012. Infarct regions were defined according to ASPECTS system on the initial head computed tomography. Discriminant function analysis was used to define specific ASPECTS regions that are predictive of the 90-day functional outcome as defined as a modified Rankin Scale score of 3–6. In addition, logistic regression was used to model the relationship between each individual ASPECT region with poor outcome; for evaluation and comparison, odds ratios, *c*-statistics, and Akaike information criterion values were estimated for each region.

**Results:**

Ninety patients with isolated M2 were included in the final analysis. ASPECTS score ≤6 predicted poor outcome in this cohort (sensitivity = 0.591, specificity = 0.838, *p* < 0.001). Using multiple approaches, we found that infarction in ASPECTS regions M3 and M6 were strongly associated with poor functional status by 90 days.

**Conclusion:**

Infarction in ASPECTS regions M3 and M6 are key predictors of functional outcome following isolated distal M2 occlusion. These findings will be helpful in stratifying outcomes if validated in future studies.

## Introduction

The site of arterial occlusion represents one of the most important factors determining outcome after anterior circulation ischemic stroke (1, 2). However, relatively little is known regarding outcome predicting variables in patients with distal middle cerebral artery (MCA) occlusion.

The Alberta Stroke Program Early CT Score (ASPECTS) was introduced to provide a structured infarct size and location analysis to aid clinical decision making (3). Its availability on initial evaluation of non-contrast head computed tomography (CT) makes it a relevant neuroimaging marker that does not require complex image post-processing. Its utility for acute treatment decision making in acute stroke patients with MCA occlusion has previously been documented (4). Specifically, stroke patients with a high pre-treatment ASPECTS are more likely to have a favorable outcome. However, because patients with distal MCA occlusion (M2) tend to have a high ASPECTS related to the sparing of subcortical tissues (5–7), a more granular understanding of the association of the ASPECTS with outcome in these patients is needed.

A better understanding of this issue is highlighted by the transformative results from the recent positive endovascular stroke trials (2, 4, 8–10). However, less than 3% of enrolled patients had an isolated M2 occlusion and the majority of patients experience a favorable outcome irrespective of endovascular therapy (4, 7, 11). Hence, identifying imaging parameters that predict poor outcome in this population may aid in the proper patient selection for therapy.

Therefore, we sought to determine whether information regarding infarct location encoded in the ASPECTS allows for defining patients at high risk for a poor functional outcome after isolated M2 occlusion in addition to the total ASPECTS score. We hypothesized that infarction in distinct ASPECTS location will be associated with a poor outcome.

## Materials and Methods

### Study Sample

We performed a retrospective analysis of consecutive acute ischemic stroke patients admitted to a single academic center from January 2010 to August 2012. Only patients with isolated M2 occlusion on admission CT-angiography (CTA) were included. This study was approved by the Institutional Review Board of the University of Massachusetts Medical School. Because this was a retrospective analysis the consent requirement was waived. Included patients have been described as part of a prior study (12).

All patients had head CT performed on presentation to the emergency room. Patient demographics, comorbidities, pre-admission medications, laboratory data, treatment modality [conservative management versus acute intervention (intravenous thrombolysis and endovascular recanalization)], and stroke etiology (according to the Trial of Org 10172 in Acute Stroke Treatment classification) (13) after completion of diagnostic evaluation, were collected on all patients. Admission National Institutes of Health Stroke Scale and modified Rankin Scale (mRS) scores were assessed at the time of presentation and at 90 days by a stroke-trained physician or study nurse certified in the mRS (12). Good outcome at 90 days was defined as mRS ≤2.

### Neuroimaging Protocol

All CT sequences were obtained on a 64-row detector Philips scanner. NCCT was performed in a non-helical mode at 120 KvP and 200 mA with data reconstruction at 5 mm axial slices. CTA was performed using 64 mm × 0.625 mm detector configuration with a pitch of 0.673, from the arch of aorta to the vertex using 120 KvP, 300 mA, and 0.5-s rotation time. Patients received 60–80 mL of Isovue 370 (Bracco Diagnostics, Princeton, NJ, USA) in the antecubital vein at a rate of 4 mL/s through a power injector followed by 40 mL saline. Three-dimensional orthogonal maximum intensity projection images were created in three planes.

### Image Review and Analysis

Image review and analysis has been described in detail earlier (12). Briefly, CT and CTA were reviewed independently by study physicians masked to clinical data, follow-up scans, patient variables, and outcomes. The M2 segment was defined as the segment extending from the bifurcation/trifurcation of the MCA to the top of the Sylvian fissure to further division (12). For the purpose of this study, we performed an infarct location analysis on the initial head CT as defined by ASPECTS system (3). These areas included the insula (I), caudate nucleus (C), lentiform nucleus (L), internal capsule (IC), superior parietal lobe (M6), precentral and superior frontal lobe (M5), anterior superior frontal lobe (M4), inferior parietal and posterior temporal lobe (M3), temporal lobe (M2), and anterior inferior frontal lobe (M1) (14). Scoring was conducted by an experienced reader (Muhib Khan) trained in ASPECTS (http://www.aspectsinstroke.com).

### Statistical Methods

All analyses were conducted using SAS Software 9.4 (SAS Inc., Cary, NC, USA). Poor outcome (mRS 3–6) was modeled with ASPECTS score using multivariable logistic regression, both as an ordinal measure and dichotomized >6 versus ≤6. Discriminant function analysis (DFA) was used to derive a single statistical function from all ASPECTS regions that distinguishes between a poor and favorable outcome; ASPECT regions were then evaluated by how well each region correlated with the derived discriminant function distinguishing between a poor and favorable outcome. DFA was used as it is more robust than multivariable logistic regression for small sample sizes. DFA was produced using the CANDISC procedure. In order to confirm and quantify the relationship between each region and outcome, logistic regression was used to model the relationship between each individual ASPECT region with poor outcome using the LOGISTIC procedure; odds ratios (OR) (effect size), *c*-statistics [area under the receiver operating characteristics curve (AUC)], and Akaike information criterion (AIC) (AIC-relative model fit) values were, therefore, estimated for each region. In addition, sensitivity, specificity, as well as positive and negative predictive values and likelihood ratios were determined for each ASPECTS region predicting poor outcome using generalized estimating equations (GEE), which allowed multiple observations (regions) to be nested within patients, using the GLIMMIX procedure. Group differences examined in Table 1 were compared using the χ^2^-square test or Wilcoxon test. A two sided *p* < 0.05 (when applicable) was considered significant and all estimates were calculated for 95% confidence interval (95% CI).

**Table 1:** **Baseline characteristics (unadjusted) of the patient sample as stratified by Alberta Stroke Program Early CT Score (ASPECTS)**.

Characteristics	ASPECTS ≤6 (*n* = 24)	ASPECTS >6 (*n* = 66)	*p*-Value
Age, years (IQR)	79.5 (67–85.5)	75.5 (62–85)	0.430
Female (%)	66.7	51.5	0.200
Admission NIHSS (IQR)	16 (6–24)	7 (4–10)	0.009
i.v. rtPA (%)	21	27	0.536
Hypertension (%)	71	82	0.259
Dyslipidemia (%)	46	61	0.211
Diabetes (%)	25	38	0.255
Prior stroke or TIA (%)	13	15	0.751
Atrial fibrillation (%)	29	32	0.810

*IQR, interquartile range, i.v., intravenous; mRS, modified Rankin Scale; NIHSS, National Institutes of Health Stroke Scale; PH, parenchymal hemorrhage Type 1 and 2; rtPA, recombinant tissue plasminogen activator; TIA, transient ischemic attack*.

## Results

Overall, 90 patients with isolated M2 occlusions were included for analysis. Among these, 66 (73%) patients had an ASPECTS of >6 and the majority of patients (69%) had a good 90-day outcome. Baseline characteristics of included patients as stratified by ASPECTS ≤6 versus >6 (15) are summarized in Table 1.

The mean ASPECTS score was 7.1, 95% CI 6.8–7.4. An ASPECTS score ≤6 was associated with poor outcome (sensitivity = 0.591, specificity = 0.838, *p* < 0.001). Logistic regression analysis indicated that for every unit decrease in ASPECTS score, the odds of poor outcome increased more than twofold (OR: 2.32, 95% CI 1.50–3.59, AUC = 0.799, *p* < 0.001). We then sought to determine whether distinct infarct regions encoded by the ASPECTS allow for defining patients at risk for a poor functional outcome. Because more than one ASPECTS region was frequently infarcted in a given patient, DFA was used to model the relationship of region on poor outcome. Table 2 summarizes each region’s relationship with poor outcome derived from the DFA whereby larger loading and coefficient values indicate a stronger relationship with the statistically derived discriminant function that distinguished between and poor and favorable outcome. In particular, infarction in M3 had the strongest relationship with the discriminant function relative to all other regions, followed by M6. The relationship with each region, alone, was then quantified using logistic regression, which indicated that infarction in M3 and M6 had statistically significant and relatively large OR (right M3 = 115, left M3 = 14.9, right M6 = 25.7, left M6 = 10.4), indicating a relationship with poor outcome. Moreover, infarction in M3 and M6 had the largest AUCs indicating superior discrimination between good and poor outcomes, relative to all other areas. M3 and M6 also had the smallest AIC values, indicating that models of poor outcome using M3 and M6 were superior to models using other ASPECTS encoded regions, i.e., the combined analysis with logistic regression and DFA indicates that infarction in cortical M3 and adjacent M6 ASPECTS regions on either side are the strongest predictors of a poor outcome after M2 occlusion. Infarction in the left M5, right M2, and right M5 should also be considered as indicators of poor outcome as they were statistically significant or approached significance relative to the remaining regions.

**Table 2:** **Discriminate function analysis, prediction, and performance of individual Alberta Stroke Program Early CT Score region infarction indicative of a poor outcome**.

Area	Discriminant function		Prediction		Performance			
	Loading	Beta	OR	95% CI	AUC	95% CI	AIC	*p*
**Right**								
M1	0.15	0.22	1.97	(0.36–10.82)	0.56	(0.40–0.71)	47.72	0.4358
M2	0.43	0.51	5.54	(1.20–25.68)	0.70	(0.53–0.87)	43.19	0.0286
M3	0.93	0.93	115	(9.32–na)	0.91	(0.81–1.00)	23.12	0.0002
M4	−0.07	0.05	0.70	(0.12–4.20)	0.53	(0.38–0.68)	48.16	0.6998
M5	0.35	0.04	4.00	(0.85–18.84)	0.66	(0.49–0.83)	44.95	0.0795
M6	0.66	0.40	25.7	(2.76–239.81)	0.81	(0.69–0.94)	34.87	0.0044
C	0.02	0.36	1.15	(0.09–14.19)	0.51	(0.40–0.61)	48.30	0.9132
IC	0.02	0.09	1.15	(0.09–14.19)	0.51	(0.40–0.61)	48.30	0.9132
L	0.03	0.45	1.17	(0.18–7.56)	0.51	(0.37–0.65)	48.29	0.8715
Insula	0.06	0.21	1.25	(0.30–5.23)	0.53	(0.34–0.71)	48.22	0.7599
**Left**								
M1	−0.19	−0.28	–	–	0.53	(0.50–0.57)	57.18	0.9728
M2	0.27	0.22	2.43	(0.62–9.56)	0.61	(0.44–0.78)	56.91	0.2038
M3	0.77	0.69	14.90	(2.75–80.29)	0.79	(0.66–0.93)	45.61	0.0017
M4	−0.19	0.00	–	–	0.53	(0.50–0.57)	57.18	0.9728
M5	0.59	0.63	12.63	(1.48–107.53)	0.73	(0.62–0.85)	49.76	0.0203
M6	0.66	0.26	10.40	(1.97–54.87)	0.76	(0.62–0.90)	48.73	0.0059
C	−0.22	−0.32	–	–	0.55	(0.50–0.59)	56.69	0.9685
IC	−0.30	0.18	–	–	0.58	(0.53–0.58)	55.14	0.9581
L	−0.19	0.18	0.38	(0.04–3.35)	0.56	(0.45–0.67)	57.66	0.3821
Insula	−0.16	−0.09	0.60	(0.16–2.27)	0.56	(0.39–0.73)	58.02	0.4524

*Loading and beta coefficients derived from discriminant function analysis, where stronger positive values relate to the function of poor outcome. Odds ratios (ORs), area under the receiver operating characteristics curve (AUC), and Akaike information criterion (AIC) are derived from logistic regression modeling, which estimate size of effect, diagnostic performance, and model fit, respectively*.

Last, each region was evaluated for diagnostic performance of poor outcome. Using GEE, we found that M3, M5, and M6 for both the right and the left had a high sensitivity and specificity (*p* < 0.01) for predicting a poor outcome (Table 3). Specifically, infarction in right M3 region was highly sensitive (0.91) and specific (0.92) of poor outcome.

**Table 3:** **Sensitivity, specificity, positive and negative predictive values, and likelihood ratios of individual Alberta Stroke Program Early CT Score region infarction for poor outcome (mRS >2)**.

Area	Sensitivity	Specificity	PPV	NPV	+LR	−LR
**Right**						
M1	0.27	0.84	0.43	0.72	1.70	0.87
M2	0.64	0.76	0.54	0.83	2.65	0.48
M3	0.91	0.92	0.83	0.96	11.36	0.10
M4	0.18	0.76	0.25	0.68	0.76	1.08
M5	0.73	0.60	0.44	0.83	1.82	0.45
M6	0.91	0.72	0.59	0.95	3.25	0.13
*C*	0.09	0.92	0.33	0.70	1.14	0.99
Internal capsule (IC)	0.09	0.92	0.33	0.70	1.14	0.99
*L*	0.18	0.84	0.33	0.70	1.14	0.97
Insula	0.46	0.60	0.33	0.71	1.15	0.90
**Left**						
M1	0.00	0.93	0.00	0.78	0.00	1.08
M2	0.64	0.58	0.28	0.86	1.52	0.63
M3	0.82	0.77	0.47	0.94	3.52	0.24
M4	0.00	0.93	0.00	0.78	0.00	1.08
M5	0.91	0.56	0.34	0.96	2.06	0.16
M6	0.82	0.70	0.41	0.94	2.71	0.26
*C*	0.00	0.90	0.00	0.78	0.00	1.11
IC	0.00	0.84	0.00	0.76	0.00	1.19
*L*	0.09	0.80	0.10	0.77	0.45	1.14
Insula	0.46	0.42	0.17	0.75	0.79	1.29

*PPV, positive predictive value; NPV, negative predictive value; +LR, positive likelihood ratio; −LR, negative likelihood ratio*.

## Discussion

The most important findings of our study are that infarction in the M3 and M6 ASPECTS region predict a poor outcome after isolated M2 occlusion. We and others have previously shown that a substantial minority of patients with M2 occlusion have a poor outcome by 3 months (7, 11, 12, 16). Our results show that the anatomical and geometric information of the ASPECTS allows for more specific identification of patients at high risk for poor outcome after isolated M2 occlusion.

The ASPECTS template for anatomical lesion mapping within the MCA territory has the advantage that it does not require elaborate volumetric image analysis and allows for identifying patients likely to have a poor outcome in the emergency setting (4, 17, 18). The dichotomized ASPECTS score has been used to predict outcome in earlier studies, which found an ASPECTS greater than 6 to 7 to be predictive of good outcome (3, 4, 17, 19). Recent multicenter cohort of M2 occlusion patients undergoing thrombectomy also showed that ASPECTS ≥6 predicts a good outcome (7). Consistent with these prior results, we found that ASPECTS ≥6 predicted a good outcome.

Using DFA, we found that in our sample, infarction in M3 and M6 locations were most predictive of a poor outcome. This is not too surprising because even relatively small infarcts in the M3 region (which includes the language centers located within the inferior parietal and posterior temporal lobes) and M6 region (which includes the primary motor cortex) may cause significant disability (20, 21) related to ensuing motor deficits and aphasia (22, 23). Of note, though infarction of subcortical structures such as lentiform nucleus and IC have been identified as predictors of outcome (20, 24, 25), we did not find such an association in our study. However, this discrepancy may be explained by the fact that our study was underpowered to detect such an association due to low number of patients with a subcortical infarction. It is possible that subcortical infarction occurred mostly in patients who originally had an occlusion of the M1-segment of the MCA [which gives rise to the lenticulostriate arteries to supply the striatocapsular region (26, 27)] and then subsequent clot migration to the M2 segment [which predominantly supplies the cerebral cortex and adjacent white matter (26–28)] by the time of CTA.

We did not find any effect on outcome based on hemispheric laterality of stroke. Earlier studies have shown an impact of hemispheric laterality on functional outcomes after ischemic stroke (29–32). We feel that smaller infarct volume and neglect might have played a role in minimizing the effect of lateralization. Further study is required to determine the effects of lesion laterality and the impact on stroke outcome. Endovascular therapy in these patients has recently been shown to improve outcomes (7). Therefore, further research is needed using an external stroke cohort evaluating our findings to improve patient selection for this particular group. We envision the development of an imaging index that incorporates assessments of ischemic core volumes and location in conjunction with markers of collateral and perfusion status. Such an index will allow for accurate and rater unbiased selection of patients for emergent recanalization therapies for M2 occlusion.

The strengths of our study relate to the analysis of a well-defined patient population based on advanced intracranial vascular imaging. We utilized multiple statistical methods in a stepwise fashion to identify the ASPECTS regions, which predict outcomes in this population. The collection of a large number of clinically relevant variables contributed to improved data quality while limiting the potential for misclassification. Limitations of our study relate to its retrospective design and relatively small sample size. However, to account for the low sample size, we used DFA instead of logistic regression alone. Williams and Titus found that, as a general rule, sample size should be about 3xP variables [in our case *p* = 10, for each group (30)] (33). In addition to mitigate the risk of DFA-related model overfitting, we also provided the results of simple logistic regression as well as repeated measures logistic regression with modeling of the different ASPECTS regions within each patient. Importantly, our models were stable as indicated by the consistent results across model solutions (sensitivity, specificity, loadings, odds ratio, area under the receiver operator characteristics curve, AIC, and *p*-Values). Further, diagnostic and therapeutic modalities were used at the treating stroke physician’s discretion, which may have introduced bias. Moreover, non-contrast CT head was used to determine ASPECTS, which might be less sensitive than newer imaging modalities. Our findings require confirmation in further, prospective studies and should only be considered hypothesis generating.

## Conclusion

In conclusion, this study shows that infarct topography as defined by ASPECTS allows for prediction of functional outcome after M2 occlusion. These findings may be helpful for stratifying outcomes if validated in other studies.

## Author Contributions

MK: study concept and design, data acquisition, statistical analysis, interpretation of data, and drafting the article. GB: statistical analysis, interpretation of data, and drafting the article. RG: data acquisition, interpretation of data, and critical revision of the manuscript for important intellectual content. BS: interpretation of data and critical revision of the manuscript for important intellectual content. NH: data acquisition, interpretation of data, drafting the article, and critical revision of the manuscript for important intellectual content.

## Conflict of Interest Statement

The authors declare that the research was conducted in the absence of any commercial or financial relationships that could be construed as a potential conflict of interest.
